# Unveiling immune checkpoint regulation: exploring the power of *in vivo* CRISPR screenings in cancer immunotherapy

**DOI:** 10.3389/fgene.2023.1304425

**Published:** 2023-12-14

**Authors:** Yuxiang Wang, Athar Khalil, Amina Kamar, Mengyan Du, Trang Dinh, Christopher McFarland, Zhenghe Wang

**Affiliations:** ^1^ Department of Genetics and Genome Sciences and Case Comprehesive Cancer Center, Case Western Reserve University, Cleveland, OH, United States; ^2^ Centre for Digital Transformation, Imperial College, London, United Kingdom

**Keywords:** CRISPR (clustered regularly interspaced short palindromic repeat)/Cas9 (CRISPR associated protein 9)-mediated genome editing, PD-1, immune check inhibitor (ICI), CTLA-4, *in vivo* CRISPR screen, invitro CRISPR screen, tumor immunotherapy

## Abstract

Immune checkpoint inhibitors (ICIs) have revolutionized cancer immunotherapy by reinvigorating antitumor immune responses, but their efficacy remains limited in most patients. To address this challenge and optimize Immune check inhibitor treatment, understanding the underlying molecular intricacies involved is crucial. The emergence of CRISPR-Cas9 technology has empowered researchers to precisely investigate gene function and has introduced transformative shifts in identifying key genes for various physiological and pathological processes. CRISPR screenings, particularly *in vivo* CRISPR screenings, have become invaluable tools in deciphering molecular networks and signaling pathways governing suppressive immune checkpoint molecules. In this review, we provide a comprehensive overview of *in vivo* CRISPR screenings in cancer immunotherapy, exploring how this cutting-edge technology has unraveled potential novel therapeutic targets and combination strategies. We delve into the latest findings and advancements, shedding light on immune checkpoint regulation and offering exciting prospects for the development of innovative and effective treatments for cancer patients.

## 1 Introduction

Immune checkpoints play a crucial role in maintaining immune system homeostasis by preventing prolonged immune cell activation and safeguarding normal tissue integrity (Emens et al., 2017). However, cancer cells exploit these checkpoints to evade immune surveillance and establish immune tolerance. To counteract this evasion strategy, immune checkpoint inhibitors (ICIs) have emerged as a transformative approach to reinvigorate antitumor immune responses (Finck et al., 2020; Sharma et al., 2023). The FDA approval of Ipilimumab (Yervoy), the first ICI targeting cytotoxic T-lymphocyte antigen-4 (CTLA-4), marked a significant milestone in cancer immunotherapy (Hodi et al., 2010). Subsequently, the approval of several anti-programmed death-1 (PD-1)/programmed death-ligand 1 (PD-L1) therapies, including Nivolumab, Pembrolizumab, Atezolizumab, Avelumab, Durvalumab, and Cemiplimab, further revolutionized the field (Hodi et al., 2010; Topalian et al., 2012; Herbst et al., 2014) Despite the remarkable clinical successes achieved with anti-CTLA-4 and anti-PD-1/PD-L1 therapies, their efficacy remains limited for most patients (Emens et al., 2017),and some individuals experience acquired resistance or immune-related adverse events (irAEs) (Schoenfeld and Hellmann, 2020). The quest to understand and overcome resistance mechanisms while optimizing the clinical efficacy of ICIs has become increasingly urgent in recent years. Addressing this challenge requires innovative approaches to elucidate the underlying molecular intricacies involved. The advent of the Clustered Regularly Interspaced Short Palindromic-associated endonuclease 9 (CRISPR-Cas9) mediated genome editing technology has revolutionized the field of genetics and emerged as a powerful tool for precisely investigating gene function. CRISPR screenings, represented by the landmark development of the genome-scale CRISPR-Cas9 knockout (GeCKO) library by Shalem et al., have introduced a transformative shift in our ability to identify key genes responsible for various physiological and pathological processes (Shalem et al., 2014). Leveraging the power of CRISPR technology, researchers have harnessed its potential to investigate novel therapeutic targets and combination strategies that can augment the effectiveness of ICIs. CRISPR screenings have proven invaluable in dissecting molecular networks and signaling pathways underlying immune checkpoint regulation, paving the way for the development of innovative cancer therapeutics (Manguso et al., 2017).

In this review, we summarize the broad methodologies and approaches involved in CRISPR screening libraries, with a particular emphasis on creating and utilizing *in vivo* CRISPR screenings within the realm of cancer immunotherapy. Through the lens of these innovative tools, we will delve into the latest findings and advancements that contribute to our understanding of cancer immunotherapy and offer exciting prospects for the development of novel and effective treatments for cancer patients.

## 2 The CRISPR-Cas9 screening strategy

While next-generation sequencing (NGS) technologies have greatly improved the understanding of genetic variants, many identified genes turned out to be “passenger gene alterations” that do not harbor any functional significance (McDaniel et al., 2013; Vogelstein et al., 2013), but can often elicit immunoediting when presented as neoepitopes. The breakthrough discovery of the CRISPR-Cas9 system by Charpentier and Doudna in 2012 fundamentally reshaped genome editing technology (Jinek et al., 2012), The CRISPR-Cas9 system enables precise modification of genomes, including gene removal, introduction of mutations, as well as gene silencing or activation (Doudna and Charpentier, 2014). This technology has been continuously modified to enhance efficiency, reduce off-target effects, and simplify delivery methods (Zhang, 2019). Large-scale genomic screening strategies have utilized the CRISPR/Cas9 system’s capability to edit multiple sites simultaneously (Shalem et al., 2014). The development of the first genome-scale CRISPR-Cas9 knockout library in 2014 enabled comprehensive screening of 18,080 human genes, facilitating negative and positive selection screening in human cells ((Shalem et al., 2014). Since then, CRISPR technology has been widely applied in various fields, including development and evolutionary biology, immunology, tissue regeneration, oncogenesis, metastasis, and drug discovery (Xu and Li, 2020).

In the landscape of genetic functional testing, CRISPR/Cas9-based screens present significant advantages over conventional RNAi or cDNA library screens, primarily owing to their superior genetic editing capability with reduced off-target effects (Gilbert et al., 2014; Doench et al., 2016). This revolutionary technology enables precise targeting of both coding and non-coding regions throughout the genome, offering unprecedented versatility (Gilbert et al., 2014; Doench et al., 2016). For loss-of-function screenings involving protein-coding genes, researchers can opt for two distinct approaches: CRISPR knockout (CRISPRko) or CRISPR interference (CRISPRi), each tailored to specific research goals (Koike-Yusa et al., 2014; Shalem et al., 2014). CRISPRi proves valuable for gene dosage studies, exploring reversible gene expression, or when working with cell lines exhibiting highly rearranged genomes. In contrast, CRISPRko stands as the preferred choice for investigating gene essentiality and achieving stable gene knockout (Koike-Yusa et al., 2014; Shalem et al., 2014).

### 2.1 Methodological insights: *In vitro* and *in vivo* CRISPR/Cas9 screenings

**FIGURE 1:**
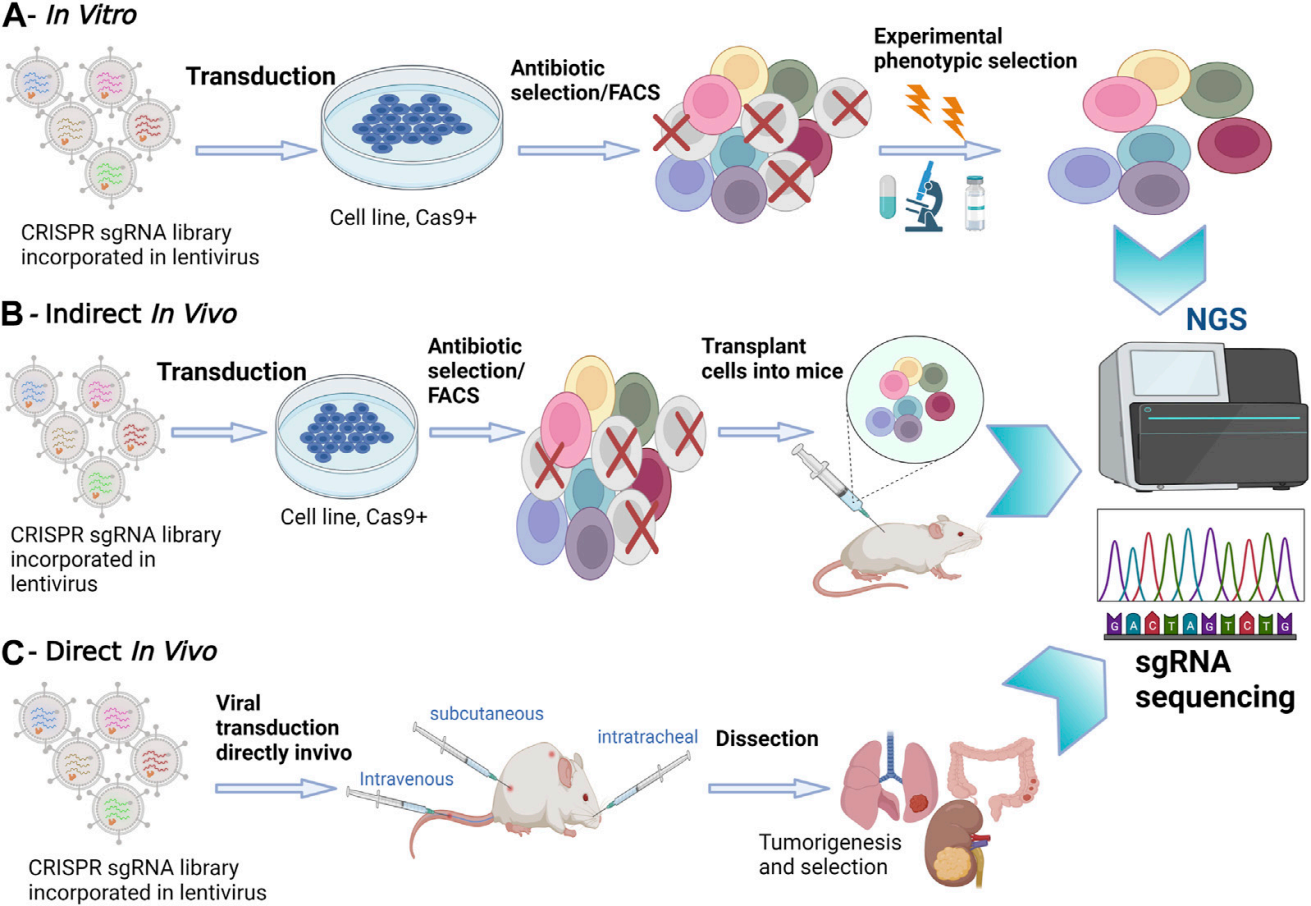
*In Vitro versus In Vivo* CRISPR/Cas9 Screenings. **(A)** *In Vitro* Screening: *In vitro* experiments take place in a controlled laboratory environment. CRISPR-Cas9 technology is applied to manipulate genes of interest in cell cultures or isolated genetic material. NGS is utilized to analyze the outcomes, providing a comprehensive view of genetic changes. **(B)** Indirect *In Vivo* Screening: In this method, CRISPR-edited cells are first cultured *in vitro*. Subsequently, these edited cells are transplanted into a living organism, often a model organism such as a mouse model. NGS is employed to monitor the changes in gene expression and genetic modifications in the host organism. This approach allows for assessing the *in vivo* impact of edited genes. **(C)** Direct *In Vivo* Screening: CRISPR-Cas9 editing is performed directly within the living organism. NGS is applied to analyze genetic changes within the host organism itself, eliminating the need for prior *in vitro* culturing. This approach provides real-time insights into gene editing effects within the organism’s natural context.

In tissue culture experiments, the use of pooled screenings employing single guide (sgRNA) libraries targeting multiple genes simultaneously has emerged as a powerful tool to attribute functional phenotypes to various gene perturbations (Doench et al., 2016). Conducting *in vitro* screening experiments is relatively straightforward, involving cell transduction with the packaged library of interest, followed by antibiotic selection. To ensure individual sgRNA expression, it is crucial to maintain a low viral load during transduction, typically ranging between 30% and 50% (Doench et al., 2016)Subsequently, NGS is employed to determine the relative sgRNA frequency across the transduced cells, effectively establishing stable cellular barcodes (Figure 1) (Doench et al., 2016). *In vitro* screenings offer the advantage of using broader libraries, facilitating comprehensive exploration of gene functions. In contrast, *in vivo* screens often necessitate more focused libraries due to increased noise and delivery challenges when targeting multiple tissues (Kuhn et al., 2021). However, *in vivo* screening offers a deeper understanding of the functional outcomes of gene perturbation, considering the influence of cell-extrinsic stimuli that can significantly impact cellular phenotypes (Doench et al., 2016).

Options for *in vivo* screening involve either transplanting CRISPRko-modified cells or directly delivering CRISPR compounds into living tissues. Transplantation entails introducing transduced cancer cells into mice and analyzing sgRNA abundance in surviving tumors through deep sequencing. However, this method has limitations in recapitulating the gradual and spatially determined interplay of local and cell-extrinsic niche factors leading to oncogenesis. Additionally, injecting a large number of cells can damage the animal’s tissues and compromise the validity of transplantation-based metastasis screenings (Chow and Chen, 2018). To address these challenges, direct *in vivo* screenings have been developed to provide a more precise recapitulation of human body conditions. Most direct *in vivo* screenings involve delivering CRISPR libraries to Cas9 transgenic mice, offering advantages in discovering genes involved in oncogenesis, cancer evolution, tumor initiation and progression (Figure 1) (Platt et al., 2014; Adamson et al., 2016). The delivery method often involves the use of viral vectors, such as lentiviruses, to introduce CRISPR components into the target cells. These screenings ensure the retention of the tumor’s native microenvironment and the natural response of the immune system to tumorigenesis. However, a limitation of direct *in vivo* screens is the relatively poor delivery efficacy of viral vectors into solid tumors (Doench et al., 2016). Despite this challenge, direct *in vivo* screens remain instrumental in unraveling the intricacies of gene function within the physiological complexities of living organisms, providing crucial insights for therapeutic developments and precision medicine.

### 2.2 *In vivo* precision: CRISPR screening studies in tumor immunotherapy advancement

Despite the significant progress of ICIs in cancer treatment, a substantial proportion of patients still do not show modification of tumor progression (Gandhi et al., 2018). The high heterogeneity and corresponding evolvability of tumors, along with the presence of multiple inhibitory immune checkpoint molecules that collectively promote immune escape, has led to the exploration of combination therapies as a future direction for tumor immunotherapy (Pauken and Wherry, 2015). Performing unbiased CRISPR screenings for potential targets to enhance the antitumor activity of ICIs can be achieved by utilizing sgRNA libraries targeting the whole genome or at least genes implicated in multiple functional classes (Li et al., 2014). However, tumor evolution is a complex and dynamic process that cannot be accurately replicated by *in vitro* CRISPR systems alone (Liu et al., 2020). *In vivo* CRISPR screenings offer several advantages for exploring candidate targets by manipulating the direct and indirect interactions between immune cells and tumor cells, providing a better representation of the immune microenvironment(Schumann et al., 2015; Manguso et al., 2017). Thus, this review exclusively focuses on the promising realm of all *in vivo* screenings that have been done in the field of tumor immunotherapy.

#### 2.2.1 CRISPR-Cas9 screenings using CRISPRko-modified cancer cells

As solid tumors can avoid detection by the various arms of the immune system or limit the extent of immunological killing, evading immune responses is recognized as a hallmark of cancers (Hanahan and Weinberg, 2011). Cancer cells escape immune surveillance by several mechanisms that fall under three major principles: a) lack of tumor antigen recognition, b) induction of immunological tolerance particularly through immunosuppressive factors, c) resistance to cell death (Fouad and Aanei, 2017; Jhunjhunwala et al., 2021). To discover potential targets that can sensitize cancer cells to immunotherapies, multiple CRISPR/Cas9-based *in vivo* screenings were conducted in cancer cells with genome-scale or focused sgRNA libraries (Table 1).

**TABLE 1:** CRISPRko screens in cancer cells.

Library name	Library size	ICI	Potential targets	Type of tumor	References
Brie mouse genome-wide library	18,748 genes	PD-1a, CTLA-4a	IFNγ	Melanoma, pancreatic, lung, renal and colon cancers	Dubrot *et al.* (2022)
Mouse GeCKO v2 genome-wide library	20,611 genes	NA	USP22	Melanoma	(M. Li et al., 2021b)
immune evasion library	2000 genes	NA	TNF	Colon adenocarcinoma	Kearney *et al.* (2018)
Disease-related immune gene library	2,796 genes	NA	Lgals2	Triple-Negative Breast Cancer	Ji *et al.* (2022)
Epigenome library	524 genes	PD-1a	TSC1/TSC2	Lung cancer	Huang *et al.* (2022)
Epigenome library	524 genes	PD-1a	Asf1a	Lung cancer	Li *et al.* (2020)
Epigenome library	850 genes	GAFCP	KDM3A	Pancreatic ductal adenocarcinoma	(J. Li et al., 2021a)
chromatin regulation	936 genes	ICB	SETDB1	Melanoma and Lewis lung carcinoma	Griffin *et al.* (2021)
Brie kinome KO library	713 genes	NA	Chek2	Glioblastoma	Dmello *et al.* (2023)
Custom library	2,368 genes	PD-1a	PTPN2	Melanoma	Manguso *et al.* (2017)
Custom library	2,368 genes	PD-1a	ADAR1	Melanoma	Ishizuka *et al.* (2019)
Tumor initiation, progression, and immune modulation Library	4,500 genes	NA	cop1	Triple-Negative Breast Cancer	Wang *et al.* (2021)

##### 2.2.1.1 CRISPR/Cas9 screenings with genome-scale sgRNA libraries

Genome-scale CRISPRko screening offers a powerful tool for investigating the connection between genotype and a specific phenotype, such as response to ICIs, in a highly efficient and parallel manner. Mouse GeCKO v2 genome-wide library and Brie mouse genome-wide library are two widely used sgRNA libraries. Dubrot et al. performed eight genome-scale screenings in mouse-transplantable tumors using the Brie mouse genome-wide library. Eight cell lines from five cancer types (melanoma, pancreatic, lung, renal, and colon cancers) were infected with the lentiviral Brie mouse library and implanted into untreated and ICI-treated immunocompetent mice, with NOD SCID mice as controls. Although the comparison between the ICI-treated and wild-type (WT) groups did not reveal a substantial number of hits for six of eight models, ICI treatment produced more significantly depleted or enriched sgRNAs than the untreated condition compared to the NOD SCID group. Comparing ICI treatment and NOD SCID groups, two pathways stood out with shared regulators among different cancer models: the Interferon (IFN) sensing and signaling pathway [Janus kinase 1 (Jak1), Jak2, Signal transducer and activator of transcription 1 (Stat1) and Interferon gamma receptor 1 (Ifngr1)] and antigen processing and presentation pathway [Transporter associated with antigen processing 1 (Tap1), Tap2, TAP-associated glycoprotein (Tapbp), Calreticulin (Calr), Protein disulfide-isomerase A3 (Pdia3), Type 1 tumor necrosis factor receptor shedding aminopeptidase regulator (Erap1), Beta-2-Microglobulin (B2m) and histocompatibility 2, T region locus 23 (H2-T23)] (Dubrot et al., 2022). On the other hand, Li et al. infected ovalbumin-expressing B16 cells (B16-OVA) with the lentiviral mouse GeCKO v2 library. The infected B16-OVA cells were then subcutaneously injected into the right flank of mice mixed with an equal number of SIINFEKL-specific CD8 + T cells (OT-I T cells). SgRNA abundance was quantified by NGS 1 month after transplanting. Ubiquitin-specific peptidase 22 (Usp22) ranked 1 and 7 from two independent repeated *in vivo* screenings, suggesting that Usp22 deficiency mediated resistance to T cell killing in B16-OVA cells (M. Li et al., 2021b). Usp22 directly interacted with Stat1, deubiquitinated it and improved its stability in melanoma cells. JAK-STAT-IFN signaling pathway highlighted in both genome-wide screenings indicated that targeting the IFN signaling pathway might be a promising strategy to sensitize cancer cells to ICIs.

##### 2.2.1.2 CRISPR/Cas9 screenings with immune-related sgRNA libraries

Immune evasion is a key characteristic of cancer (Hanahan and Weinberg, 2011). While the innate and adaptive immune system can normally recognize and eliminate tumors, certain cancer cells possess mechanisms to escape immune detection or limit immunological killing (Mittal et al., 2014). To identify the genes and signaling pathways involved in immune evasion, Kearney et al. first conducted a genome-wide CRISPR screening *in vitro* using MC38-OVA cells incubated with activated OT-I T cells and MC38 cells incubated with natural killer (NK) cells in the presence of IgG or anti-PD-1 (Kearney et al., 2018). The top-ranked enriched genes in both groups included tumor necrosis factor receptor superfamily member 1A (Tnfrsf1a) and Caspase 8 (Casp8) in the TNF signaling pathway, Jak1 and Stat1 in the IFN-γ signaling pathway, as well as Tap1 and B2m related to antigen presentation (Kearney et al., 2018)In a subsequent experiment, the top 2000 sgRNAs from both screens were cloned into a custom library, named the immune evasion library, and introduced into MC38-OVA cells expressing Cas9. These cells were then implanted into recipient NSG mice, followed by injection of OT-I T cells after tumor formation (Kearney et al., 2018). Sequencing of harvested tumors revealed enrichment of sgRNAs targeting genes involved in IFN-γ signaling (Stat1), antigen presentation (Tap1), and TNF signaling [Casp8, tnfrsf1a, and 2-aminoethanethiol dioxygenase (Ado)] (Kearney et al., 2018). Deletion of key genes within the TNF signaling, IFN-γ signaling, and antigen presentation pathways provided protection for tumor cells against CD8^+^ T cell-mediated killing and impaired antitumor immune responses *in vivo* (Kearney et al., 2018). To uncover the immune-related genes in the antitumor or protumor process that might indicate potential therapeutic strategies, Ji et al. designed and generated a mouse sgRNA library corresponding to 2,796 human disease-related immune genes, which termed the disease-related immune gene library (DrIM library). DrIM-transduced 4T1-Cas9 cells were subcutaneously transplanted into immunocompetent BALB/c mice and immune-deficient NOD-NGP mice. After 14 days, the tumors were harvested for high-throughput sgRNA library sequencing. 227 candidate genes were dichotomized into immune escape genes and immune surveillance genes and constructed into a mini-DrIM sgRNA library. A second round *in vivo* screening was performed in BALB/c mice, BALB/c-Nude mice only lacking T cells, CB-17 scid mice lacking T cells and B cells, and NPG mice lacking T cells, B cells, and NK cells. By comparing different groups, the screening provided chances to reveal candidates responsible for responsiveness to specific immune cells. Five genes were identified as potential regulators of immune surveillance in all screening settings, among which galectin 2 (Lgals2) induced the increased number of tumor-associated macrophages and resulted in the immunosuppressive microenvironment (Ji et al., 2022). The result provided a theoretical basis for LGALS2 as a potential immunotherapy target.

##### 2.2.1.3 CRISPR/Cas9 screenings with epigenetic sgRNA libraries

Epigenetic modulation genes play crucial roles in cancer biology, and accumulating evidence indicates their involvement in modulating the tumor immune microenvironment (TIME) and regulating the antitumor immune response (Zhu et al., 2015). To comprehensively assess cell-intrinsic epigenetic regulators of tumor immunity, Li et al. conducted an *in vivo* CRISPR screen using an epigenetic-focused sgRNA library targeting 524 epigenetic regulators (Li et al., 2020). By comparing sgRNAs recovered from subcutaneous xenografts derived from Kras^G12D^/Trp53^−/−^ mouse lung cancer cells in mice treated with control IgG and anti-PD-1 antibody, several hits were identified, including Tap2, Jak2, Stat1, Catenin Beta 1 (Ctnnb1), Anti-Silencing Function 1A Histone Chaperone (Asf1a), Mitogen-Activated Protein Kinase 3 (Mapk3), and TSC Complex Subunit 1 (Tsc1), that influenced the sensitivity to anti-PD-1 treatment. Loss of Asf1a induced immunogenic macrophage differentiation in the TIME and enhanced T cell activation in combination with anti-PD-1(Li et al., 2020). Another gene hit, Tsc1, was further investigated by Huang et al., who found that TSC1/2 deficiency upregulated PD-L1 expression, making TSC1/2-deficient lung cancer cells more responsive to anti-PD-1 therapy (Huang et al., 2022). Moreover, Griffin et al. employed a sgRNA library targeting chromatin genes to identify ICI sensitizers. They transduced the library into B16 melanoma and Lewis lung carcinoma (LLC) cells, which were then transplanted into mice for tumor-cell vaccination and PD-1 blockade or combination PD-1/CTLA-4 blockade (Griffin et al., 2021). The H3K9-methyltransferase SET Domain Bifurcated Histone Lysine Methyltransferase 1 (Setdb1) emerged as the top-ranked sensitizer in both B16 and LLC models, as its loss led to the de-repression of transposable elements (TEs) capable of generating major histocompatibility complex class I (MHC-I) peptides and triggering T cell responses (Seoane et al., 2019). Another epigenetic library targeting 850 epigenetic factors and RNA-binding factors was applied by Li et al. in the subcutaneous pancreatic ductal adenocarcinoma (PDAC) model. By comparing sgRNA abundance in GAFCP (gemcitabine, G; nab-paclitaxel, A; anti-CD40 agonist, F; anti-CTLA-4, C; and anti-PD1–1, *p*) treatment group and control group, they identified Lysine Demethylase 3A (Kdm3a) as a potent epigenetic regulator of immunotherapy response in PDAC (J. Li et al., 2021a). These discoveries emphasize the promising opportunity of directing attention towards epigenetic factors to boost the effectiveness of immune checkpoint inhibitors. This can be achieved not only by regulating PD-L1 expression but also by reshaping the immune microenvironment.

##### 2.2.1.4 CRISPR/Cas9 screenings with kinome sgRNA libraries

As kinases are a major drug target and a major control point in cell behavior, the kinase has also been the target of large-scale functional genomics with CRISPRko screenings and drug discovery efforts, especially in cancer therapeutics (Workman, 2005). To investigate the contribution of glioma cell-intrinsic kinases in T cell recognition, GL261 cells were intracranially implanted into WT and CD8 deficient C57BL/6 mice after they had been transfected with a CRISPRko library for all 713 known kinases (Dmello et al., 2023). Among the kinase KO clones depleted in WT mice relative to the CD8 deficient mice, which contributed towards resistance to CD8 T-cell-mediated killing, checkpoint kinase 2 (Chek2) had the most depleted sgRNA. Mechanistically, loss of Chek2 enhances antigen presentation, STING pathway activation, and PD-L1 expression in mouse gliomas, supporting Chek2 as a promising target for enhancement of response to immune checkpoint blockade therapy in glioblastoma (GBM) (Dmello et al., 2023).

##### 2.2.1.5 CRISPR/Cas9 screenings with other custom sgRNA libraries

A commonly employed approach to overcome the high cost and achieve comprehensive coverage of sgRNAs in whole-genome *in vivo* CRISPR screens is the utilization of custom sgRNA libraries (Li et al., 2014). Wang et al. utilized a custom murine CRISPR-Cas9 knockout (MusCK) library containing 5 sgRNAs for each of the over 4,500 genes implicated in tumor initiation, progression, and immune modulation. They performed *in vivo* CRISPR screens in 4T1 cells implanted in syngeneic BALB/c mice. A subsequent library (MusCK 2.0) focused on 79 candidate genes identified in the primary screen, with 8 sgRNAs per gene Significant depletion of immune evasion mediators (Cd274/Pd-l1), components of the IFN-γ signaling pathway [Jak1, Jak2, Stat1, and Interferon regulatory factor 1 (Irf1)], an E3 ubiquitin ligase (Cop1), and an oncogenic transcriptional activator [Tripartite Motif Containing 24 (Trim24)] was observed (Wang et al., 2021). Further investigation of Cop1 inhibition revealed reduced macrophage-associated chemokine secretion, decreased tumor macrophage infiltration, and synergy with ICI through polyubiquitination and proteasomal degradation of the CCAAT/enhancer-binding protein (Cebpδ) (Wang et al., 2021). In another study by Manguso et al., lentiviral vectors encoding 9,872 sgRNAs targeting 2,368 genes were used to engineer B16 melanoma cells expressing Cas9. These cells were transplanted into T cell receptor (Tcra)−/− mice or WT mice treated with GVAX and PD-1 blockade. The enrichment of five genes involved in sensing and signaling through the IFNγ pathway (Stat1, Jak1, Ifngr2, Ifngr1, and Jak2) in immunotherapy-treated mice suggested their role in MHC-I presentation upregulation. Further investigation confirmed that Protein Tyrosine Phosphatase Non-Receptor Type 2 (Ptpn2) loss activated IFN-γ signaling and increased antigen presentation (Manguso et al., 2017). Following that, Ishizuka et al. observed a significant depletion of Adenosine deaminase acting on RNA 1 (Adar1)-targeting sgRNA in tumors of immunocompetent mice treated with GVAX and PD-1 blockade. Loss of ADAR1 overcame resistance to PD-1 checkpoint blockade by restoring antigen presentation. The absence of ADAR1 reduced A-to-I editing of interferon-inducible RNA species, leading to melanoma differentiation-associated protein 5 (MDA5)-mediated immune microenvironment inflammation through the secretion of Interferon β (Ishizuka et al., 2019).

#### 2.2.2 CRISPR-Cas9 screenings in CRISPRko-modified T cells

T cells play a central role in the adaptive immune response and immunotherapy. Cytotoxic CD8^+^ T cells are responsible for destroying virus-infected cells and tumor cells. Matthew et al. designed and generated a lentiviral CRISPR vector, which contains a sgRNA expression cassette and Thy1.1, a surface antigen marker for thymocytes, resulting in a more robust efficiency in determining transduction efficiency by fluorescence-activated cell sorting (FACS) (Table 2) (Dong et al., 2019). They isolated native CD8^+^ T cells from OT-I, Cas9 mice, infected the T cells with genome-scale mouse KO library, and then transferred these T cells into Recombination activating gene 1 (Rag1)-deficient mice bearing E0771-OVA transplanted tumors (Dong et al., 2019). By harvesting tumors with tumor-infiltrating lymphocytes and analyzing the sgRNA frequency, they re-identified canonical immunotherapy targets such as PD-1 and T cell immunoglobulin and mucin-domain containing-3 (Tim-3), along with genes that have not been characterized in T cells like DEAH-Box Helicase 37 (Dhx37). Dhx37 suppressed effector functions, cytokine production, and T cell activation by modulating nuclear factor kappa-light-chain-enhancer of activated B cells (NF-kB) pathway (Dong et al., 2019). Ye et al., in the same group, applied the Sleeping Beauty transposon system to enable efficient randomized gene and regulatory-element knockout in primary murine T cells and genomic integration of the sgRNA cassette for screen readout (Ye et al., 2019). They focused on a mouse surface and membrane protein-encoding gene library targeting 1,685 genes and demonstrated that adoptive transfer of CD8^+^ T cells with Pdia3, Mannoside Acetylglucosaminyltransferase 5 (Mgat5), Epithelial Membrane Protein 1 (Emp1) or Lymphocyte-activation gene 3 (Lag3) gene editing enhanced the survival of GBM-bearing mice in both syngeneic and T cell receptor transgenic models (Ye et al., 2019). Similar strategies were applied by other researchers to conduct *in vivo* CRISPR screens in CD8^+^ T cells with different libraries. Targeting Zinc finger CCCH-type containing 12A (ZC3H12A), which encodes the ribonuclease REGNASE-1 was revealed to program long-lived effector T cells for cancer therapy by screening with a metabolic library targeting 3,017 genes(Wei et al., 2019). Rc3h1 encoding the E3 ubiquitin ligase Roquin-1 was found to be the strongest repressor of CD8^+^ T cell expansion by a genome scale CRISPR screen(Zhao et al., 2021). Surprisingly, the Fli1 (Friend leukemia integration 1) protooncogene was identified to improve effector T cells differentiation and protective immunity in cancer by screen with a library targeting 120 transcription factors (Chen et al., 2021). Multiple components of the mammalian canonical BRG1/BRM-associated factors were discovered to be essential for the differentiation of activated CD8^+^ T cells into effector T cells by screening with a library targeting 337 epigenetic regulators (Guo et al., 2022). Another chromatin-remodeling complex SWI/SNF (SWItch/Sucrose Non-Fermentable) was identified by screen with a library targeting 220 epigenetic regulators, indicating that chromatin-remodeling complex is a potential target to regulate differentiation of T cells(Baxter et al., 2023).

**Table 2:** CRISPRko screens in T cells

Library name	Library size	Potential targets	References
Genome-scale mouse KO library	19,674 genes	Dhx37	Dong *et al.* (2019)
Mouse surface and membrane protein-encoding gene library	1,685 genes	Pdia3	Ye *et al.* (2019)
Metabolic library	3,017 genes	Regnase-1	Wei *et al.* (2019)
Genome scale library	19,674 genes	Roquin	Zhao *et al.* (2021)
Transcription factor library	120 genes	Fli1	Chen *et al.* (2021)
Epigenetic regulator library	337 genes	cBAF	Guo *et al.* (2022)
Epigenetic regulator library	220 genes	SWI/SNF	Baxter *et al.* (2023)

#### 2.2.3 Direct CRISPR-Cas9 screenings

Because the immune microenvironment within a subcutaneously implanted tumor model diverges significantly from that found in a tumor in its autochthonous (natural) location, the advantage lies with *in situ* CRISPR-Cas9 screens. The primary essence of *in situ* CRISPR-Cas9 screens revolves around prompting the transformation of ordinary cells into tumorous ones within their native context and subsequently employing CRISPR-Cas9 technology to modify the genomes of these cells. Wang et al. developed a CRISPR-mediated genetically engineered mouse model (CRISPR-GEMM) of liver cancer by utilizing an adeno-associated virus (AAV)-CRISPR vector to drive autochthonous liver tumorigenesis in fully immunocompetent mice through pooled mutagenesis (Wang et al., 2020).(Table 3). The vector contained a liver-specific thyroxine-binding globulin (TBG) promoter driving Cre recombinase expression, along with two sgRNA expression cassettes. The Cre recombinase thus can induce Cas9 and firefly luciferase expression in the liver of the Lox-stop-Lox (LSL)-Cas9, LSL-Fluc mouse. As for the two sgRNAs, one targeted Trp53 to induce tumorigenesis, while the other served as a backbone for cloning and expressing specific sgRNAs targeting the top 49 most frequently mutated tumor suppressor genes in pan-cancer TCGA dataset (Wang et al., 2020). Comparing the anti-CTLA4-treated mice with the PBS-treated mice, the mutation frequencies of B2m, Glucocorticoid receptor DNA-binding factor 1 (Grlf1), BCL6 Corepressor (Bcor), and Kdm5c were significantly increased, while the mutation frequency of AT-Rich Interaction Domain 1A (Arid1a) was significantly decreased. Similarly, comparing anti-PD1-treated mice with PBS-treated mice, the knockout of B2m, Grif1, Von Hippel-Lindau Tumor Suppressor (Vhl), Cyclin-Dependent Kinase Inhibitor 1B (Cdkn1b), and Bcor was associated with anti-PD1 resistance, whereas knockout of Kmt2d, Arid1a, Ring Finger Protein 43 (Rnf43), and ATRX Chromatin Remodeler (Atrx) was linked to anti-PD1 responsiveness(Wang et al., 2020). Loss-of-function mutations in Lysine Methyltransferase 2D (*KMT2D*) were found to potentiate anti-PD1 checkpoint immunotherapy by enhancing immune infiltration in the tumor microenvironment. This was achieved through multiple mechanisms, including increased DNA damage and mutation burden, activation of TEs, elevation of IFNγ-stimulated antigen presentation, and upregulation of myeloid-recruiting cytokines (Wang et al., 2020). Dervovic et al. designed a similar vector, LV-sgRNA-Cre-OVA, to induce tumorigenesis in mouse lung tissue and conduct a CRISPR-Cas9 screen. They administered the lentiviral sgRNA library generated by LV-sgRNA-Cre-OVA vector at postnatal day 2 to the lungs of LSL-Kras^G12D^ or LSL-Braf^V600E^ mice(Dervovic et al., 2023). The Cre recombinase activated the expression of Kras^G12D^ or LSL-Braf^V600E^ in the lung tissue and induced lung cancer. OVA was applied to increase efficient antigen presentation and induce immune responses. OT-I T cells were injected in the mice via the tail-vein and anti-PD-1 treatment, anti-CTLA4 treatment or IgG control was administered 4 weeks after lung tumor induction by LV-Cre-OVA inhalation. These mice were then immunized with OVA-peptide emulsified in Complete Freund’s adjuvant on day 1 and primed with OVA/Incomplete Freund’s adjuvant emulsion on day 7 post adoptive cell transfer to stimulate specific OT-I T cell responses (Dervovic et al., 2023). sgRNAs were quantified at 6.5 weeks after tumor induction. They recovered the known immune evasion factors Stat1 and Serpin Family B Member 9 (Serpinb9) and identified the cancer testis antigen ADAM Metallopeptidase Domain 2 (Adam2) as an immune modulator, whose expression is induced by Kras^G12D^ and further elevated by immunotherapy (Dervovic et al., 2023).

**TABLE 3:** *In situ* CRISPRko screenings.

Library name	Library size	ICI	Potential targets	Type of tumor	References
Immune cytolytic activity	5,573 genes	PD-1a, CTLA-4a	Serpinb9, Adam2	Lung cancer	Dervovic *et al.* (2023)
Tumor suppressors	49 genes	PD-1a, CTLA-4a	Kmt2d	Liver cancer	Wang *et al.* (2020)

## 3 Concluding remarks and future perspectives

In conclusion, the application of CRISPR/Cas9 technology within the intricate landscape of immunology offers a tantalizing pathway towards advancing the realm of immunotherapy and presents a promising avenue for enhancing ICIs efficacy, as it enables the identification of novel targets for cancer treatment.

Furthermore, the continuous advancements and application of CRISPR/Cas9 technologies such as those that pairs CRISPR-ko with lineage tracing via random barcodes hold significant potential if combined with studies that tackle the mechanisms of immunomodulatory drugs (Rogers et al., 2017). This is a promising field to revolutionize cancer treatment, offering more precise and effective therapies for patients. Apart from the CRISPRko screens covered in this review, numerous *in vitro* screenings utilizing CRISPRa and CRISPRi have unveiled potential targets capable of enhancing CAR-T efficacy (Ye et al., 2022), mediating T cell stimulation (Schmidt et al., 2022), or acting as potential drivers of cancer resistance to NK and T cell-mediated cytotoxicity (Joung et al., 2022; Lee et al., 2023). However, it is noteworthy that the *in vivo* screening results for CRISPRa and CRISPRi libraries in this field are yet to be identified.

Despite the immense potential of CRISPR/Cas9 high-throughput screening in improving immunotherapy efficacy, certain safety and efficacy concerns remain, impeding its full translation into clinical settings. The main challenges facing the use of CRISPR/Cas9-based treatments include the ethical concerns, the identification of a proper delivery technique, the occurrence of off-targeting modifications, and the possibility of causing autoimmune disease (Rasul et al., 2022). Therefore, it is crucial to leverage extensive screening, molecular, and clinical data to deepen our understanding of tumor immunity and expedite progress in the field of cancer therapy. Through diligent research and collaboration, we can usher in a new era of personalized and targeted cancer treatments.
